# The high cost of half-hearted breastfeeding promotion in Germany

**DOI:** 10.1186/s13006-014-0022-5

**Published:** 2015-04-11

**Authors:** Elien Rouw, Elizabeth Hormann, Veronika Scherbaum

**Affiliations:** Im Wasserbett 7, Bühl, 77815 Germany; European Institute for Breastfeeding and Lactation (ret.), Neusser Str. 866, Cologne, 50737 Germany; Institute for Social Science in Agriculture, Gender and Nutrition, University of Hohenheim, 430b, Schloß, Museumsflügel, Stuttgart, 70593 Germany

**Keywords:** Breastfeeding, Prevention, Support, Education, Breast milk, Economic value

## Abstract

The economic value of breastfeeding to the society at large is under researched and its importance as a preventive public health strategy is underestimated. What little research there is indicates that considerable savings would accrue from following the WHO/UNICEF advice to breastfeed exclusively for six months and continue breastfeeding along with complementary foods for two years or more. Despite relatively high breastfeeding initiation in Germany, neither exclusive breastfeeding nor breastfeeding duration come close to international recommendations. Breastfeeding is mostly regarded as a woman’s personal choice and the government has been slow to engage in breastfeeding promotion, support and research. Some structures in Germany do offer support for breastfeeding women – including the growing number of Baby Friendly Hospital Initiative (BFHI) certified hospitals and a comprehensive maternity leave policy. However, the costs of breastfeeding are mostly borne by the mothers and those for breastfeeding training mostly by the individual health care workers or hospital, while the health insurance companies and society-at-large are profiting from the financial savings from exclusive and long-term breastfeeding. Factors which might improve breastfeeding rates and duration in this country include broad expansion of and financial support for both BFHI hospitals as well as training for the health care personnel who support the mother-infant dyad during the breastfeeding period.

## Introduction

Breastfeeding is the normal, species-specific way to feed an infant. It is far more than just nutrition. In the last few decades, scientific research has made clear that breastfeeding protects the infant against both infectious and non-infectious diseases. It enhances the bonding process between mother and child and influences the maternal hormonal system, affecting both short-term and long-term health positively. An overview of these beneficial health effects can be found in the American Academy of Pediatrics policy statement, “Breastfeeding and the Use of Human Milk” [1]. It is also noted here that “infant nutrition should be considered a public health issue and not only a lifestyle choice”. A breast-milk substitute is infant nutrition of acceptable quality, but entails health risks for both mother and child, such as an increased risk of infectious diseases, obesity and some cancers. Thus breastfeeding is generally seen as “desirable behavior” and both health care workers and governmental institutions stress the importance of breastfeeding support. Most countries have accepted the recommendation by UNICEF and WHO to have a National Breastfeeding Committee [2]. In many countries, committees have also been established to guide the Baby-Friendly Initiative, with more or less success. However, in most countries, breastfeeding is still seen as merely a mother’s personal decision. Support is something that has to be requested and paid for by the mother herself. Therefore, many mothers don’t meet their own expectations of breastfeeding [3]. Mostly, they have concerns about their own health and/or that of their babies or about the processes around breastfeeding, for which they cannot find adequate help.

## Review

### Breastfeeding and economics

Breastfeeding as an economic value has, in the past, either been taken for granted or ignored. Even though interest in breastfeeding has been on the rise, the idea that breastfeeding has an economic value and that not breastfeeding is connected with high costs for society is relatively new. In 1992, the value of all human milk produced in Australia was calculated at around $1.9 billion US (at $50/l) [4]. Had the breastfeeding rates in Australia at that moment matched UNICEF/WHO recommendations, this would have had a value of approximately 1% of the GDP. Ironically, when a cow produces milk, the GDP goes up [5] but when a woman produces milk, the GDP falls [6], which clearly shows the folly of overlooking the economic value of breastfeeding. The value of breast milk in milk banks has been calculated very recently by a German hospital at about 52€/liter which includes compensation for milk donors as well as transport, laboratory and personnel costs [7].

In an overview by J. Weiner [8] the costs of not breastfeeding in the USA were calculated for three diseases (otitis media, gastroenteritis and necrotizing enterocolitis). A decade later, these calculations were expanded [9] with newer data from the report by the Agency for Healthcare Research and Quality (AHRQ) [10]. This study looked at hospitalization for necrotizing enterocolitis, otitis media, gastroenteritis, lower respiratory tract infections, atopic dermatitis, sudden infant death syndrome, childhood asthma, childhood leukemia, type 1 diabetes mellitus, and childhood obesity and came to the conclusion that if, within a one year birth cohort, 90% of US mothers could comply with the medical recommendations for 6 months exclusive breastfeeding, the country would save $13 billion and prevent an excess 911 deaths for this cohort [9]. Further evidence, especially on chronic illnesses, can be found in a meta-analysis from Australia [11].

In 2012, Renfrew and colleagues [12] calculated the costs of not breastfeeding in Great Britain. In this overview, the outcomes for four childhood diseases (gastrointestinal disease, respiratory disease, otitis media and necrotizing enterocolitis) and also one maternal disease (breast cancer) for a one-year birth cohort were considered. The authors considered a far lower level of breastfeeding (75% exclusively breastfeeding children at discharge from the neonatal unit, 45% exclusive breastfeeding at 4 months), and only the four childhood diseases mentioned, but found that even this could save over 17 million pounds annually. Furthermore, if 50% of the mothers of a yearly birth cohort, who did not breastfeed, were to have breastfed for 18 months over their lifetimes, there would have been 865 fewer cases of breast cancer in this cohort, resulting in a 21 million pound reduction in health care costs.

In these reviews, the well-known effects of breastfeeding on the intellectual, social and emotional development of the child are not taken into account. There are further hidden costs for not breastfeeding as well. For employers, it has been calculated that a lesser degree of breastfeeding will lead to more parental absence due to illness of the children and, thereby, to higher costs [13],[14]. For decades, many nations have provided substantial, sometimes hidden, subsidies to artificial feeding, while not funding breastfeeding support. Most recently, China’s government has also begun to provide subsidies ($4.9bn) for the production of breast milk substitutes to compete against foreign brands [15],[16] instead of supporting breastfeeding. Currently, the fastest growing infant formula market is in Asia. This is again associated with high long-term costs for society. Most economic calculations do not consider the ecological value of breastfeeding, which leads to lower costs for society: lower CO2 production by comparison to the manufacture of breast milk substitutes, bottles and teats, transportation and waste [17]. So it is clear, that the numbers given are only the very minimal calculations of the costs of not breastfeeding.

### The situation in Germany

There is little data on the breastfeeding situation in Germany. More than a decade ago, the SuSe Study [18] was undertaken to measure the breastfeeding rates in Germany. Further data come from the KiGGs Study, a large study on health and lifestyle of babies and young children, which also collected data on breastfeeding [19], from a study to determine the breastfeeding situation in Bavaria [20] and, most recently, a study conducted in the city of Freiburg [21] Looking at these scanty, mostly non-representative and difficult to compare data, it seems that the breastfeeding situation in Germany is a bit better than in some other European countries. Initiation of breastfeeding in all studies is quite high, around 90%, although in some sub-populations, especially among socially disadvantaged and smoking women, the initiation rate is much lower. As in most Western countries, the breastfeeding rate in Germany drops quickly after the first few days. At three months, around 40% of babies are exclusively breastfeeding [18] and, at six months, around 22% are exclusively breastfeeding. The Freiburg study [21], which looks at any breastfeeding, cites relatively high rates – 74% at 3 months, 61% at 6 months, and 28% at 12 months. All of these breastfeeding rates, although they probably give an overly positive estimate for the population at large, are, nonetheless, clearly below the WHO recommendations. Unfortunately, up to the present time, systematic monitoring of breastfeeding rates has not taken place due to lack of (governmental) funding. This makes a precise estimate of the breastfeeding situation in Germany impossible. There are also no systematic studies on the economic aspects of breastfeeding in Germany. A conservative calculation, based on the incidence of otitis media [22], the costs of otitis media [23] and prevention of 25% of cases of otitis media through breastfeeding, showed an annual savings of about €11 million [24].

### Breastfeeding support and cost effectiveness

In Germany, although there is agreement - in theory - on the value of breastfeeding and it has been clearly said, that breastfeeding is the feeding method of choice for an infant, this statement is more a mantra than a real commitment by the government or health services to act. Breastfeeding is mostly regarded as a mother’s personal decision. Nevertheless, there are a few major instruments to support breastfeeding: breastfeeding support in hospitals, breastfeeding support in the community, and maternity leave programs. There is also a law on breastfeeding support in the work place [25]. There is even a law in Germany that was designed to protect the public from misleading advertisements from the infant formula industry (Säuglingsnahrungswerbegesetz). It protected breastfeeding with a declaration on infant formula tins reading “breastfeeding is superior to infant formula”, regulated advertising and defined penalties for violations of the law [26]. This law has been largely overtaken by EU regulations and since no penalty is defined, it is not effective anymore.

#### Breastfeeding support in the hospital

Baby-friendly hospitals clearly contribute to increased breastfeeding rates [27]. About 17% of births in Germany take place in a Baby-Friendly hospital and every year new hospitals are added [20]. However, the total costs for the initial certification of a baby-friendly hospital are around 60,000 to 80,000€. This process usually takes about two to four years and includes staff training and the certification process. Recertification is required after three years and this process, as well as the mandatory continuing education for the personnel involved, again costs another 3,000 to 4,000€ [28],[29]. Although hospitals can profit from this, due to increased client satisfaction and probably more births/year, the cost can be a significant barrier to the certification process [30]. There are no governmental instruments to encourage the certification of more baby-friendly hospitals and reimbursement stays the same for a hospital, with or without certification. Furthermore, due to cost reductions, there is a general understaffing of maternity wards, leading to less breastfeeding support, which can be time consuming in the first few days after birth.

#### Breastfeeding support after discharge from hospital

Breastfeeding support at home is provided mainly by midwives and every woman is entitled to this support for 8 weeks after birth and even longer, when there is a medical indication. However, many midwives are not educated in breastfeeding support, although such support clearly makes a difference in breastfeeding rates [31]. The services of a lactation consultant are not paid for by general insurance companies, so have to be paid by the mother herself. Physicians (general practitioners, gynecologists, pediatricians) are generally not trained in breastfeeding medicine and their support, or lack thereof, is mostly shaped by their own (sometimes negative) experiences of breastfeeding [32]. This is regrettable because better counseling would lead to improved clinical outcomes [33]. As the Freiburg study [21] notes: “Following the normally brief hospital stay and the midwife’s support of and advice to the mother, the advice of gynecologists and neonatologists with experience in breastfeeding is also urgently required”. A major barrier to better counseling is that regular training of health care professionals in breastfeeding education is mostly not a part of medical curricula. Breastfeeding programs have to be financed by hospitals or individuals themselves, unlike education on artificial infant nutrition, which is offered for free by the infant formula industry.

In Germany there are only a few community-based breastfeeding support programs for mothers. There is a network of peer-group breastfeeding supporters, but the education of these lay counselors is also mostly financed by the counselors themselves. The Baby-Friendly Community Initiative has not yet been established in Germany.

It may be assumed, that, as in other countries, the sharp drop in breastfeeding rates after the first few days of life is due to lack of breastfeeding support and is a major reason that women do not achieve their own breastfeeding goals [3].

On the other hand, maternity leave, which is very important for breastfeeding support, is well-established in Germany: 14 weeks fully paid maternity leave (6 weeks before the birth, 8 weeks after the birth or 12 weeks for preterm or multiple birth), 12 months parental leave with 65% of the mother’s salary (partly paid for by health insurance companies and partly by employers) and unpaid parental leave until the child is 3 years old. However, more and more women are not taking the maternity leave to which they are entitled for fear of disadvantages in the work place for their careers and, in the long run, even for their pensions, which in Germany are mostly based on lifetime earnings. A longer maternity leave does have a positive influence on breastfeeding rates and, thus, on lowering health care costs. However, as long as maternity leave is seen as a hindrance to career development, the negative consequences of taking maternity leave will be carried by mothers [34].

In the work place, breastfeeding women are entitled to two 30 minute breaks per 8 hour day to breastfeed or pump their milk. This time is counted as working time and has to be paid as such [35]. However, in this law there are no specific recommendations for the provision by the employer of private places to pump or of storage places for pumped milk. This is again the responsibility of the mother, to pro-actively seek a place to pump and provide the equipment.

When mothers choose to breastfeed, this often leads to personal disadvantages for them, while society profits greatly, without having made any investment in supporting it. When mothers choose not to breastfeed, this often leads to personal advantages for them, along with huge costs for society. As Hall Smith comments: “Role conflict and strain lead to behavioral choices that may stem more from time and resource constraints than from personal preferences” [34]. Up until now, there have been no cost-effectiveness studies in Germany on breastfeeding interventions, as is usual for vaccinations [36]. Considerable amounts of money from the health care system are invested in immunization programs on the presumption that, by preventing disease, this financial investment is a cost-effective measure. This also means that parents don’t have to make a financial contribution to this preventive measure, since vaccinations for infants are offered without charge. The same should apply for the investment in breastfeeding support programs. Breastfeeding promotion should not impose economic and other costs on women [11],[37].

## Conclusion

Breastfeeding is the normal, species-specific nutrition for human infants. Not breastfeeding not only leads to a greater burden of disease for both mother and child, but also to high economic costs, which have to be paid for by society. However, by contrast to disease prevention through vaccinations, individual support of breastfeeding is not seen as a governmental task. Much of the professional breastfeeding support is offered in Baby-Friendly hospitals, at considerable cost for these hospitals. At this moment, no free formal breastfeeding training curricula for nurses, midwives or physicians are available. Training in breastfeeding medicine for health care workers must be paid for by hospitals or by the health care workers themselves, again at considerable cost, by contrast with education on artificial baby food, which is offered at no charge by the infant food industry.

When there are problems with breastfeeding, support by health care workers with a background in lactation support, such as International Board Certified Lactation Consultants (IBCLC) also has to be paid for by the mothers themselves. At present, many health care providers don’t have the training to support these mothers adequately. Furthermore, longer breastfeeding until the age of 2 years and more – as recommended by the WHO - can have a negative influence on a mother’s career chances and on the amount of her pension when she retires.

As long as words are not followed up by deeds, and praise for breastfeeding is not followed up by financial support of breastfeeding initiatives, many mothers will not be able to achieve their own breastfeeding goals nor will the UNICEF/WHO recommendations for breastfeeding be achieved by most of them. With proven preventive effects for acute and chronic diseases, breastfeeding is cost effective as a disease prevention measure. Investment in breastfeeding support would certainly pay for itself.

## Authors’ information

**Elien Rouw** is a Dutch physician, living and working in Germany. She is a member of the German National Breastfeeding Committee and an active member of several national and international breastfeeding organizations. She regularly trains health care workers in clinics on breastfeeding. She is the co-author of a book on breastfeeding for health care workers and the director of an educational center for breastfeeding.

**Elizabeth Hormann** is a psychotherapist with long-time interest in breastfeeding. She was an assessor for the Baby-Friendly Hospital Initiative (BFHI) for 20 years, leading assessments and training local assessors in Europe and the Middle East. Over that same period she taught in the German-language training program for the qualifying exam for International Board Certified Lactation Consultants (IBCLC). Currently she writes, edits and translates professional articles and books related to breastfeeding.

**Veronika Scherbaum** is a Senior Researcher at the Institute for Social Sciences in Agriculture, Gender and Nutrition at the University of Hohenheim. As a diploma graduate in Nutrition Science, she earned her PhD at the Institute of Biological Chemistry and Nutrition, University of Hohenheim, in the field of dietary management of malnutrition among children in Ethiopia. In addition, she earned an MSc in Mother and Child Health at the University of London, has published numerous nutrition and breastfeeding papers in international journals, and is the editor and co-author of a German-language book on breastfeeding, nutrition in early childhood and reproductive health (Stillen: frühkindliche Ernährung und reproduktive Gesundheit).
